# Proton pump inhibitors in inpatients: Are we getting it right? A retrospective analysis

**DOI:** 10.5339/qmj.2024.60

**Published:** 2024-11-11

**Authors:** Gowri Karuppasamy, Yousef Mohammad Saleh Yahia, Jessiya Veliyankodan Parambil, Shanima Ismail, Mohammed Ibn-mas’ud Danjuma

**Affiliations:** 1 Department of Medicine, Hamad Medical Corporation, Doha, Qatar *Email: kgowri207@gmail.com; 2 Department of Gastroenterology, Hamad Medical Corporation, Doha, Qatar

**Keywords:** Proton pump inhibitors, acid-suppressive therapy, hypomagnesemia, renal impairment

## Abstract

**Background:**

Proton pump inhibitors (PPIs) are commonly prescribed to hospitalized patients, but many of these prescriptions may not be based on evidence-based indications. It’s important to understand that inappropriate prescribing of PPIs can lead to unnecessary medications and financial burdens. Unfortunately, there are not many recent studies exploring how often PPIs are prescribed and if they are being prescribed appropriately.

**Objective:**

The study aimed to assess the appropriateness of PPIs use among hospitalized patients. It evaluated the indications for PPIs use and determined whether the use of PPIs in hospitalized patients is justified or not.

**Setting:**

The study was conducted at Hamad General Hospital, a tertiary academic healthcare center in the state of Qatar.

**Methods:**

A retrospective observational study with 201 subjects, was conducted in general internal medicine wards at a tertiary hospital. Physician documentation and inpatient and outpatient medication prescriptions were analyzed for PPIs exposure.

**Main outcome measures:**

The appropriateness of exposure to PPIs is determined based on international recommendations.

**Results:**

Of 533 hospitalized patients who were not critically ill, 201 (37.7%) were prescribed PPIs. The study found that 65.2% of the patients had no valid indication for PPIs exposure. Furthermore, 18% of patients were inappropriately prescribed stress ulcer prophylaxis with PPIs even though they had a low risk for the development of ulcer disease. After discharge, 82.6% of patients were prescribed PPIs, with the most common indication (43%) being gastrointestinal ulcer prophylaxis.

**Conclusion:**

This study sheds light on the issue of overutilization of PPIs, specifically in non-critically ill hospitalized patients. It highlights the unnecessary continuation of PPI prescriptions at discharge and emphasizes the importance of physicians reevaluating PPI prescriptions periodically to ensure they are still necessary and discontinuing them when possible to avoid unwanted consequences.

## Impacts on Practice

• The uncritical prescription of inpatient PPIs may lead to unnecessary and continued prescription in outpatient care, resulting in potential for exposure to adverse outcomes.

• It is important to develop institutional protocols and educational interventions to restrict PPIs use for justified indications.

## 1. Introduction

Proton pump inhibitors (PPIs) are widely prescribed to hospitalized patients.^1-3^ However, there is a growing concern that PPIs are often misused and not prescribed based on evidence-based indications.^4,5^ This leads to unnecessary and continued prescriptions in outpatient settings, resulting in polypharmacy and increased medication costs.^5-7^ Although PPIs are generally safe, there are potential adverse effects associated with long-term use, including an increased risk of renal disease, hypomagnesemia, fractures, myocardial infarction, ischemic stroke, gastric cancer, dementia, Clostridioides difficile infection, community-acquired, and hospital-acquired pneumonia.^8-11^.

This study aimed to evaluate the indications and appropriateness of PPIs use among patients admitted to the general internal medicine service at a tertiary hospital. The goal is to help physicians make informed therapeutic decisions and inform therapeutic commissioners.

## 2. Methods

A retrospective cross-sectional study was conducted at the Weill Cornell Medicine affiliated-Hamad General Hospital, a tertiary center in Doha, Qatar. Electronic medical records of consecutive patients admitted to general internal medicine wards over 1 month were reviewed retrospectively. Physician documentation and inpatient and outpatient medication prescriptions were analyzed for PPIs. Case record forms were designed on Excel spreadsheets for data entry.

PPIs exposure was defined as administering any PPIs medication for at least 7 days. Appropriate PPIs exposure was determined based on the US Food and Drug Administration (FDA)-approved list of accepted indications, as well as recommendations from the Canadian Medical Association (CMA).^12,13^ Off-label use was considered inappropriate use of PPIs.

We included patients admitted to general internal medicine services who were prescribed PPIs. The following patients were excluded: patients who were not receiving PPIs, those who were admitted to the intensive care unit or transferred from the intensive care unit to the medical ward, those with incomplete records, patients with documented underlying hyper-secretory syndromes, pregnant women, and patients on concomitant medications known to attenuate the action of PPIs.

The following variables were extracted from the patient’s electronic records: patient demographics (age and gender), admitting diagnosis and other diagnoses, as well as patient’s medications, focusing on the use of aspirin, clopidogrel, anticoagulants, non-steroidal anti-inflammatory drugs (NSAIDs), and steroids. Documentation was reviewed for the presence of a history of upper GI bleed, peptic ulcer disease (PUD), and gastroesophageal reflux disease (GERD). Additional variables regarding inpatient PPIs use were extracted, including name, dose, route, and frequency of PPIs administration. Discharge prescriptions were also analyzed for PPIs, including indication and prescription duration.

The local ethical committee approved the study: Medical Research Center of Hamad Medical Corporation, Doha, Qatar, approval number MRC-01-19-022.

Data were analyzed using the statistical package for the social sciences (SPSS) software (version 20.0). Data were summarized and presented as means and standard deviation, or median interquartile range as appropriate, for continuous variables and numbers/percentages for categorical variables. A generalized estimating equation (GEE model) with compound asymmetry estimated the incidence of PPIs use between wards.

## 3. Results

Electronic medical records of 533 patients admitted to Hamad General Hospital under the general internal medicine department during 1 month were reviewed, of whom 201 patients were on PPIs (37.7%). The demographic characteristics of the study population are described in Table 1. The mean age of the patients on PPIs was 57.22 ± 18.58.

Significant comorbidities of the patients in the PPIs group included hypertension (41.2%), diabetes mellitus (38.3%), chronic kidney disease (29.6%), and coronary artery disease (21.4%). Fifty percent of the patients in the PPIs group were on aspirin, 19.9% on steroids, and 10.9% on other NSAIDs.

93.5% of the patients were on oral PPIs, 5.5% were receiving a twice-daily dose, and the rest were on a once-daily regimen. We also noted that 65.2% of the 201 patients were already on PPIs before admission, and for 95.5% of the patients, it was continued throughout the hospital stay (Table 2).

### 3.1. Indications for appropriate and inappropriate PPIs use

Of the patients exposed to PPIs, 65.2% had no valid reasons to use them (Table 3). The most common appropriate reason for using PPIs was to provide gastroprotection when taking NSAIDs in the high-risk group, which accounted for 32.9%. On the other hand, the most common inappropriate reason for using PPIs was gastropathy prophylaxis associated with corticosteroids, antiplatelets, or anticoagulants without any risk factors for ulcer disease. Other common appropriate reasons for PPIs usage were gastric and duodenal ulcers with documented exacerbations within the last 3 months and symptomatic GERD, both accounting for 24.7% each. In 23.6% of the patients, no valid reasons for using PPIs could be identified.

### 3.2. Prescription of PPIs upon discharge

The vast majority (82.6%) of patients were prescribed PPIs upon discharge. Prescription durations varied from 2 months (21.7%) to 1 year (13.8%), which was assessed by review of electronic medical records. The most common reason for prescription was the prophylaxis of peptic ulcer disease associated with corticosteroids, antiplatelets, or anticoagulants in patients without any increased risk of the disease (43%, *n* = 71). However, there was no detectable reason for prescription in 16.4% of patients (*n* = 27).

## 4. Discussion

Our study revealed that 65.2% of patients were exposed to PPIs without a clinical need, as per the guidelines established by the FDA and CMA.^12,13^ While PPIs are highly effective in managing acid-related disorders,^14,15^ concerns have arisen regarding their overutilization in conditions where their benefits have not been conclusively proven.^4,5,16^ These findings align with those of previous studies. A survey conducted by Grant et al. reported that 40% of hospitalized patients were inappropriately started on PPI therapy, with 70% of these patients continuing PPI use 6 months later.^17^ A more recent study further highlighted that 51.92% of patients were already misusing PPIs at the time of admission, with this figure increasing to 57.25% by discharge.^18^ Additionally, 18% of the patients in our study were unnecessarily prescribed stress ulcer prophylaxis despite having a low risk of gastrointestinal bleeding. This is consistent with a review indicating that 20–25% of general medical patients receive acid suppression for stress ulcer prophylaxis without risk factors for bleeding.^19^ Another observational study found that 27.75% of surgical inpatients received prophylactic PPIs inappropriately during the perioperative period.^20^ In our cohort, 82.6% of the patients were discharged on PPIs, with ulcer prophylaxis being the most common indication (43%), even though the risk of ulcer disease was low. This trend of overprescribing inpatient PPIs can lead to their continued use in outpatient settings without reassessing their necessity.^7,21,22^ Similarly, Winter et al. discovered that 25% of primary care patients were prescribed PPIs for unapproved reasons, primarily for conditions initiated during hospitalization.^23^ Numerous studies have linked prolonged PPI use with various adverse effects. Our patient group, which included individuals with comorbidities, such as hypertension, diabetes, chronic kidney disease, and coronary artery disease, experienced these potential adverse effects more frequently. It is crucial to emphasize that PPIs should only be prescribed when necessary. The American Gastroenterological Association (AGA) strongly advises that the long-term use of PPIs should be regularly reviewed to ensure that the lowest effective dose is administered.^24^ In 2022, AGA released the Best Practice Advice, recommending regular PPI use reviews for all patients, with the responsibility of lying with primary care providers. Discontinuation should be based on the absence of a clear indication for use rather than concerns regarding adverse effects.^25^ This study marks a significant milestone in our organization’s efforts to enhance patient care. As the first analysis of this nature within our institution, it serves as a crucial step towards developing and implementing protocols to improve patient outcomes. Public education on the risks associated with chronic PPI use could help address this issue with information disseminated via websites, social media, and healthcare events. Additionally, healthcare providers should engage in discussions with patients regarding long-term PPI use. A multidisciplinary approach and patient engagement are key to addressing this challenge; however, the retrospective study design has certain limitations. Being a cross-sectional study, there is a potential for prevalence bias, although our findings are consistent with those of other studies. Despite its short duration, the study’s sample size was adequate for providing a general understanding of prescription practices. Further prospective controlled trials are needed to better understand the long-term implications of inappropriate PPI use.

## 5. Conclusion

This study has brought to light the issue of overuse of PPIs in hospitalized patients who are not critically ill and the unnecessary continuation of PPIs prescriptions upon discharge. This puts patients at risk of various adverse outcomes. To address this problem, it is essential to develop institutional protocols and educational interventions that restrict PPIs usage to only justified indications. Physicians should also periodically re-evaluate the long-term PPIs prescription to minimize potential adverse effects.

## Authors’ Contributions

GK was involved in study concept development, liaison with regulatory agencies, study design, data acquisition and organization, data analyses, and manuscript preparation. JVP, YMSY, and SKI contributed to data collection, data analyses, and manuscript writing. MD was involved in data organization, data analyses, manuscript preparation, and provided expert advice.

## Acknowledgments

We acknowledge the contribution of Dr. Syed Irfan Alam to study concept development and Mr. Prem Chandra for statistical analyses.

## Declarations and Conflict of Interest Statement

None of the authors have any conflict of interest to declare.

## Figures and Tables

**Table 1.** Demographic Characteristics of the Study Population.

**Variable**	
**Age (years)**	
Mean ± SD	57.22 ± 18.58
Median (range)	64.00 (17–92)
**Gender N (%)**	
Male	139 (69.2%)
Diabetes mellitus	77 (38.3%)
Hypertension	83 (41.2%)
Chronic kidney disease	59 (29.6%)
Coronary artery disease	42 (21.4%)
**Medication use**	
Aspirin	100 (49.8%)
Clopidogrel	31 (15.4%)
Anticoagulants	22 (10.9%)
NSAIDs	22(10.9%)
Steroids	40 (19.9%)
**Risk factors**	
History of GERD	18 (9.0%)
History of upper GI bleed	14 (7.0%)
History of peptic ulcer disease	9 (4.5%)

**Table 2.** Disposition of Individual PPIs Analogue Usage.

**Variable**	**Number (%) (*n* = 201)**
**PPI agent**	
Esomeprazole	60 (29.8%)
Lansoprazole	28 (13.9%)
Omeprazole	1 (0.5%)
Pantoprazole	85 (42.3%)
Rabeprazole	27 (13.4%)
**Route of administration**	
Oral	188 (93.5%)
Intravenous	13 (6.5%)
**Frequency of administration**	
Once daily	190 (94.5%)
Twice daily	11 (5.5%)
**Continuation of PPIs from outpatient to inpatient**	
Patients on PPIs before hospital admission	131 (65.2%)
PPIs continued throughout hospital stay	192 (95.5%)

**Table 3.** Indications for appropriate and inappropriate PPIs use.

**Variable**	**Number (%) (*n*=201)**
PPIs use appropriate	70 (34.8%)
PPIs use inappropriate	131 (65.2%)
**Among those who had appropriate indication for PPIs use (*n* = 70)**	
Gastric and duodenal ulcer with documented exacerbations within the last 3 months	18 (24.7%)
Symptomatic GERD	18 (24.7%)
Healing or maintenance of erosive esophagitis	5 (6.8%)
Prophylaxis for gastropathies associated with NSAIDs	24 (32.9%)
Others, including acute upper GI bleed, use of dual antiplatelet agents, and aspirin use in patients aged >60 years	8 (11.0%)
**Among those who had inappropriate indication for PPIs use (*n* = 131)**	
No discernible indication	30 (23.6%)
Low-risk stress ulcer prophylaxis for non-critically ill medical patients	23 (18.1%)
Prophylaxis of PUD associated with corticosteroids, antiplatelets, or anticoagulants without concomitant NSAID use	67 (52.8%)
History of gastrointestinal (GI) bleed, or PUD for more than 3 months without ongoing complications or exacerbations	2 (1.6%)
Others, including abdominal pain, pancreatitis, and oesophageal varices	5 (3.9%)
